# Diversity of the Antibody Response to Tetanus Toxoid: Comparison of Hybridoma Library to Phage Display Library

**DOI:** 10.1371/journal.pone.0106699

**Published:** 2014-09-30

**Authors:** Mahsa Sorouri, Sean P. Fitzsimmons, Antonina G. Aydanian, Sonita Bennett, Marjorie A. Shapiro

**Affiliations:** Laboratory of Molecular and Developmental Immunology, Division of Monoclonal Antibodies, Center for Drug Evaluation and Research, Food and Drug Administration, Bethesda, Maryland, United States of America; National Cancer Institute, NIH, United States of America

## Abstract

Monoclonal antibodies are important tools in research and since the 1990s have been an important therapeutic class targeting a wide variety of diseases. Earlier methods of mAb production relied exclusively on the lengthy process of making hybridomas. The advent of phage display technology introduced an alternative approach for mAb production. A potential concern with this approach is its complete dependence on an *in vitro* selection process, which may result in selection of V_H_-V_L_ pairs normally eliminated during the *in vivo* selection process. The diversity of V_H_-V_L_ pairs selected from phage display libraries relative to an endogenous response is unknown. To address these questions, we constructed a panel of hybridomas and a phage display library using the spleen of a single tetanus toxoid-immunized mouse and compared the diversity of the immune response generated using each technique. Surprisingly, the tetanus toxoid-specific antibodies produced by the hybridoma library exhibited a higher degree of V_H_-V_L_ genetic diversity than their phage display-derived counterparts. Furthermore, the overlap among the V-genes from each library was very limited. Consistent with the notion that accumulation of many small DNA changes lead to increased antigen specificity and affinity, the phage clones displayed substantial micro-heterogeneity. Contrary to previous reports, we found that antigen specificity against tetanus toxoid is encoded by both Vκ and V_H_ genes. Finally, the phage-derived tetanus-specific clones had a lower binding affinity than the hybridomas, a phenomenon thought to be the result of random pairing of the V-genes.

## Introduction

Therapeutic monoclonal antibodies (mAbs) have proven to be effective therapies in the treatment of a variety of cancers and autoimmune diseases. Because of this success, mAbs and related products are the fastest growing class of human therapeutics [1] and their use is being developed for other chronic indications such as osteoporosis [2] and hypercholesteremia [3]. MAbs also play an important role in biomarker validation and diagnostic assays [4].

Historically, mAbs, including therapeutic mAbs, were generated by making hybridomas from mice immunized with the antigen of choice. The V_H_ and V_L_ regions were then engineered to produce chimeric or humanized mAbs that retain the specificity and affinity of the murine mAb, but impart more favorable properties of human antibodies, such as lower immunogenicity, improved half-life and effector function. Today, human mAbs are the predominant class of mAb entering clinical studies [5].

The two most commonly used methods for production of human mAbs are hybridomas generated from mice expressing human antibody genes and phage display technologies [6]. Antibody production by hybridoma technology relies on immortalization of B-cells resulting in production of antibodies with cognate V_L_ and V_H_ gene pairs. On the other hand, antibody production by phage display technology leads to random pairing of V_L_ and V_H_ genes which may or may not represent physiological pairing configurations [7]–[10].

Despite utilization of random V_L_ and V_H_ gene pairs, phage display technology has gained popularity for development of therapeutic mAbs because it is a relatively simple process and requires less antigen and time compared to standard hybridoma technology [8], [11]. Additionally, phage display technology is able to produce antibodies against a wide range of antigens, including weak or non-immunogenic, self, cell surface, and toxic antigens, which are difficult to develop by immunizing mice [8], [12]. Phage display also allows for production of human antibodies without the need for humanized mice or concerns of Epstein-Barr virus or other potential pathogens present in human antibody-producing cell lines. Successful construction of phage display libraries derived from B cell pools from human donors or synthesized from human V-gene sequences has been reported [12]–[15]. At this time, three FDA-approved therapeutic mAbs are derived from phage display libraries.

A potential downside of antibody production using phage display technology is the lack of a “normal” selection process. V_H_ -V_L_ pairs are randomly cloned into phage display vectors and undergo antigen selection *in vitro*. This selection process is distinct from the *in vivo* process where V_H_ -V_L_ pairs expressed on developing B cells undergo negative or positive selection events prior to antigen exposure. Although it has been argued that the processes of affinity selection by panning and screening for antigen-reactive clones mimic the processes of clonal selection and expansion utilized by the mammalian immune system [8], it is important to keep in mind that the lack of negative selection in a phage display library may result in selection of antibodies that would have been eliminated *in vivo*. Such antibodies might have cross-reactivity for both endogenous antigens and the antigen of choice and would not be useful for therapeutic purposes. Moreover, any unusual idiotype which elicits a strong immunogenic response has the potential to lose its effectiveness and may result in adverse events directly or in the form of circulating immune complexes.

In this study, we compared the diversity of the immune response generated by standard hybridoma technology to that of a phage display library. We chose tetanus toxoid (TT) as our model antigen because it is complex and contains multiple epitopes, which are expected to result in a diverse antibody response. The spleen of a single TT-immunized, BALB/c mouse was used for making a hybridoma library and phage display library. We expected greater V_L_ and V_H_ gene diversity in the selected phage display antibodies, but to our surprise, TT-specific antibodies produced by the hybridomas were much more diverse than those isolated from the phage display library, and there was little overlap among the V_L_ and V_H_ genes found in each library. TT-specific phage clones contained a considerable amount of micro-heterogeneity, which suggests that improvements in specificity and affinity of an antibody to a certain antigen are most likely the result of many small changes [16]. In contrast to previous reports [17], [18], we show that antigen specificity of antibodies against TT is encoded by both Vκ and V_H_ genes. The TT-specific phage clones had overall lower binding affinities than the TT-specific hybridoma clones, which may be a result of non-cognate pairing of Vκ and V_H_ genes [18].

## Results

### Ig Gene Diversity in Hybridoma-Produced Anti-TT mAbs

The diversity of the anti-TT mAbs was assessed by DNA sequencing the Vκ and V_H_ genes from 21 TT-specific mAbs. Table 1 shows the V(D)J gene usage and specificity (tetanus fragment C of the heavy chain (Hc) or light chain (TT-LC)) for each antibody. There were twelve unique Vκ genes from 7 families, with three members of the Vκ4 family recurring in 6 hybridomas. Eighteen unique V_H_ genes from 5 families were identified, with most hybridomas expressing V_H_ genes from the 7183, Q52 and J558 families. There were only two sets of clonally related hybridomas; 18-96 and 26 utilized a 19-25/Jκ4 V_L_ and VH36-60.a2.90/DSP2.5/JH2 V_H_ and hybridomas 6 and 14 utilized a kf4/Jκ5 V_L_ and VHQ52.a27.79/DSP2.5/JH2 V_H_. There was one recurrent pair; hybridomas P4T29115 and 5 both used the VH7183.a15.24 and ce9 V region genes, but different D and J genes. There were several hybridomas that used different genes from the same Vκ and V_H_ families, but these often had different specificities. For example, hybridomas 18–96 and 7–55 both use genes from the VH36-60 family and the Vκ19 L chain family, but bind TT-LC and Hc, respectively. Eleven TT-binding mAbs were specific for Hc, while three of the independent (not clonally related) non-Hc-binding mAbs were specific for TT-LC, one Hc-binding mAb (hybridoma 16) also bound TT-LC and three independent mAbs were specific for TT epitopes not found on either Hc or TT-LC.

**Table 1:** V(D)J Gene Usage and Binding Kinetics of the Anti-TT Hybridomas.

Hybridoma	V_H_	J_H_	D_H_	Vκ	Jκ	Hc	TT-LC	K_a_ (1/Ms)	K_d_ (1/s)	K_D_ (M)	Anti-IgG1 Capture (RU)	Rmax
1-92	J558.33	JH2	DSP2.10	bv9	Jκ2	Y	N	8.8×10^4^	2.0×10^−4^	2.3×10^−9^	92	86
13.4	J558.12	JH3	N/A	12–44	Jκ4	Y	N	1.9×10^5^	6.1×10^−4^	3.2×10^−9^	115	114
17	J558.16.106	JH2	DFL16.1	23–48	Jκ4	Y	N	ND	ND	ND	-	-
12	J558.17	JH2	DFL16.1	ap4	Jκ5	ND	N	5.6×10^4^	2.1×10^−4^	3.8×10^−9^	266	120
P394	J558.45	JH2	DSP2.11	bv9	Jκ4	Y	N	1.0×10^5^	6.0×10^−4^	6.0×10^−9^	97	48
P141.2	J558.52	JH3	N/A	kn4	Jκ5	Y	N	1.3×10^5^	4.6×10^−4^	3.5×10^−9^	50	18
18-96^1^	VH36-60.a2.90	JH2	DSP2.5	19–25	Jκ4	N	Y	9.5×10^4^	2.3×10^−4^	2.4×10^−9^	120	25
26^1^	VH36-60.a2.90	JH2	DSP2.5	19–25	Jκ4	N	Y	8.5×10^4^	2.8×10^−4^	3.3×10^−9^	102	20
7–55	VH36-60.a6.114	JH3	N/A	19–17	Jκ1	Y	N	1.5×10^5^	8.3×10^−4^	5.5×10^−9^	80	105
P429.26	VH7183.14	JH1	DSP2.5	kn4	Jκ5	Y	N	2.6×10^4^	1.1×10^−3^	4.2×10^−8^	117	71
13–65	VH7183.a10.15	JH1	DSP2.5	23–48	Jκ4	N	Y	8.4×10^4^	3.4×10^−4^	4.0×10^−9^	62	60
P4T29115^2^	VH7183.a15.24	JH4	DSP2.10	ce9	Jκ1	N	N	ND	ND	ND	-	-
5	VH7183.a15.24	JH2	DFL16.1	ce9	Jκ2	N	N	1.1×10^5^	6.7×10^−4^	6.1×10^−9^	112	109
22	VH7183.a30.50	JH2	DSP2.9	8–24	Jκ5	Y	N	7.7×10^4^	7.3×10^−4^	9.5×10^−9^	134	64
P4T3166	VH9.12	JH4	DSP2.13	ap4	Jκ4	Y	N	6.7×10^4^	3.1×10^−4^	4.6×10^−9^	129	96
21	VH9.2	JH2	DFL16.1	kf4	Jκ4	N	N	9.3×10^4^	4.0×10^−4^	4.3×10^−9^	82	68
16	VHQ52.a19.61	JH4	DSP2.10	bb1	Jκ1	Y	Y	1.1×10^5^	2.0×10^−4^	1.8×10^−9^	104	33
24	VHQ52.a2.4	JH4	DSP2.9	12–41	Jκ1	Y	N	8.6×10^4^	1.6×10^−4^	1.9×10^−9^	128	92
28	VHQ52.a24.72	JH2	N/A	12–41	Jκ1	N	Y	2.0×10^5^	5.4×10^−4^	2.7×10^−9^	127	106
6^3^	VHQ52.a27.79	JH2	DSP2.5	kf4	Jκ5	N	N	1.1×10^5^	2.3×10^−4^	2.1×10^−9^	130	113
14^3^	VHQ52.a27.79	JH2	DSP2.5	kf4	Jκ5	N	N	9.4×10^4^	2.9×10^−4^	3.1×10^−9^	155	112

ND = not determined.

1 Hybridoma 18–96 and 26 are clonally related.

2 P4T29115 was not evaluable on the IgG1-coated Biacore sensor because it is an IgG2a.

3 Hybridoma 6 and 14 are clonally related.

### Ig Gene diversity in the *E.coli* Phagemid Library and the Expanded Library

In order to ensure the coverage and amplification of a majority of Vκ genes [19]–[21], we aligned the sequence of our Vκ primers to the sequences of all Vκ genes and confirmed primer coverage of the majority of Vκ genes (86/95) with two or fewer mismatches. Only a subset of the BALB/c V_H_ gene sequences are available, thus performing a similar primer analysis for our V_H_ primers was not possible. Therefore, we relied on a V_H_ primer set that was previously used to construct BALB/c phage display libraries [22]. All the hybridoma sequences can be successfully amplified by the V_H_ and Vκ primers and none of the hybridoma sequences contain restriction sites used for cloning into the pCES vector.

Inherent bias in PCR could lead to a limited set of Vκ and V_H_ genes, as well as over-representation in the library of one or more of genes favored by the reaction conditions. Therefore, it was important to demonstrate a representative diversity of Vκ and V_H_ families and family members to ensure a diverse set V_H_/Vκ pairs for selection on TT. Diversity of the phagemid library was assessed at multiple levels. First, the Vκ and V_H_ sequences were assessed in 50 randomly selected clones after initial cloning into pCES and transformation into XL1-Blue cells and prior to expansion of the library. Twenty-two distinct Vκ and 14 distinct V_H_ genes were identified from eleven Vκ families and five V_H_ families, respectively (data not shown). Analysis of the Vκ sequences showed that the library contained both germline sequences, as well as hypermutated sequences. Two sequences (V_H_ 7183 and Vκ12-44) were overrepresented among these 50 clones, but overall, the unexpanded library was diverse for both H and L chains.

Because only a small aliquot of the phagemid library was expanded for phage production, we next checked the diversity of the expanded library in *E. coli*. Sequence analysis of 15 clones from the expanded library revealed the presence of 9 Vκ families represented by 12 genes and 5 V_H_ families represented by 9 genes. The presence of other Vκ and V_H_ families not detected by sequencing was assessed by PCR using gene-specific primers (Table S1) based on germline V-gene sequences obtained from IgBLAST [23] or IMGT [24]. Overall, all Vκ families, except dv36, which is a single gene family, and all V_H_ families, except V_H_12, VH3609N, and V_H_15, which are also single gene families, were detected in the unselected library.

### Fab Expression in the *E.coli* Phagemid Library

We also assessed Fab expression to address the possibility that specific Vκ and V_H_ sequences or V_H_/Vκ pairs are toxic for phage growth and would therefore, not be available for antigen selection. First, SS320 *E. coli* were infected with unselected phage and the resulting individual colonies were isolated and induced to express Fab. Fab expression was assessed by ELISA and showed that 34/376 colonies (9%) were capable of expressing Fab. A majority of the library Fabs did not induce well in SS320 *E. coli* using IPTG induction from the pCES phagemid, however, comparison of V_H_/Vκ sequences from Fab secretors and clones that did not secret Fabs, but contained both V_H_ and Vκ inserts did not show any apparent bias in V_H_/Vκ gene usage. Secretors utilized 23 different Vκ genes from 11 Vκ families (Vκ1, 2, 4, 9, 12, 19, 21, 23 24 and, 32) and 15 different V_H_ genes from 5 V_H_ families (7183, Q52, 36–60, J558, VH9). Non-secretors utilized 17 different Vκ genes from 8 Vκ families (Vκ1, 2, 4, 8, 9, 19, 21, and 23) and 12 different V_H_ genes from 4 V_H_ families (7183, Q52, 36–60, J558).

In a second experiment, phage was rescued at small scale from 172 individual clones from the pCES unexpanded stock library. An anti-Fab ELISA demonstrated that 60% of the clones produced phage bearing Fabs, which matches the percent of clones in the library that are known to contain both V_H_ and V_L_ sequences.

### Ig Gene Diversity of the Antigen-selected Phage Display Library

**Figure 1:**
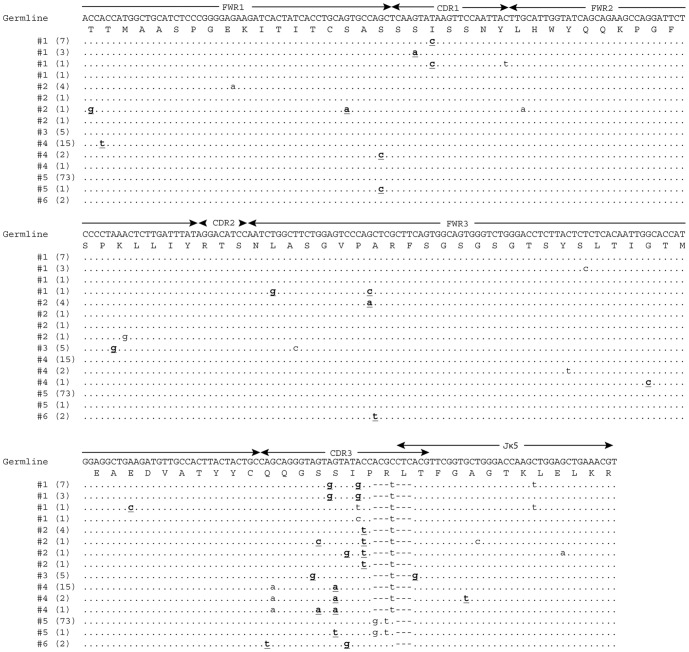
Representative IgAT analysis for V_L_ clonotypes. Fifteen unique kf4/Jκ5 DNA sequences were segregated into 6 clonotypes using IgAT. Germline sequence was obtained by manually juxtaposing the available germline kf4 gene (GenBank:AJ231229.1) and Jκ5 (GenBank:V00777.1) sequences. Numbers on the left side of the aligned sequences indicate the clonotypes determined by IgAT. The numbers in parentheses indicate the number of replicates that were isolated for each unique sequence. Sequences marked with same number are potentially clonally related. Sequence delineation is based on the IMGT system. Replacement mutations are shown in bold underlined font.

In order to maximize the potential diversity and percentage of TT-positive clones recovered after selection, the selection parameters were varied (Table 2). Additionally, separate selections were performed using TT-LC and Hc, as they represent less complex antigens compared to toxoid. No antigen-specific clones were isolated from the TT-LC selection or the selection that was done for 15 minutes on TT (Table 2, Selections 7 and 2, respectively). Combined, approximately 13% (201/1500) of the total phage clones screened from the fifth output phage pools of various selections on TT or Hc were antigen-specific. Vκ-gene usage was highly restricted to the Vκ4 (kf4), Vκ9 (ce9, cw9) and Vκ19 (19–15) families (Table 3). There were independent kf4 gene rearrangements to Jκ2, Jκ4, and Jκ5 while the ce9, cw9 and 19-15 were always rearranged to Jκ1, Jκ4 and Jκ5, respectively (Table 3). However, multiple unique sequences were detected for each Vκ/Jκ rearrangement, except 19–15/Jκ5. For example, within kf4/Jκ5, fifteen unique sequences were identified and each unique sequence was isolated multiple times (Figure 1, Table 3). In total, 38 unique Vκ/Jκ sequences were identified: 15 for kf4/Jκ5, 8 for kf4/Jκ4, 6 for kf4/Jκ2, 4 for cw9/Jκ4, 4 for ce9/Jκ4 and 1 for 19–15/Jκ5.

**Table 2:** Selection Parameters.

Selection Parameters	Selection 1 (TT)	Selection 2 (TT)	Selection 3 (TT)	Selection 4 (TT)	Selection 5 (TT)	Selection 6 (H_C_)	Selection 7 (TT-LC)
1Starting Amount of Phagemid Library	10 µL	50 µL	50 µL	50 µL	50 µL	50 µL	50 µL
Amount of Antigen/Well	0.1 µg	1 µg	1 µg	1 µg	1 µg	1 µg	1 µg
2Fraction of Phage Used in Round One	10%	100%	100%	100%	100%	100%	100%
3Fraction of Phage Used in Rounds 2–5	2.5%	10%	10%	10%	10%	10%	10%
Selection Time	120 min	15 min	120 min	120 min	120 min	120 min	120 min
# of clones	25	0	75	62	12	27	0
Wash Buffer	0.05% PBST	0.05% PBST	0.05% PBST	0.05% PBST	0.5% PBST	0.05% PBST	0.05% PBST
4# of washes	1–10	1–10	1–10	1–5	1–5	1–5	1–5
5Washing procedure	10X*	10X*	10X*	5X	5X	5X	5X

1 Indicates volume of the XL1-Blue library glycerol stock used for starting the culture from which phage particles were rescued.

2 In Selection 1, 100 µL of the 1 mL rescued phage library (10%) was divided between two antigen-coated wells. In Selections 2–7, the entire volume of the rescued phage pool (1 mL, 100%) was divided between four antigen-coated wells.

3 In Selection 1, 50 µL of the 2 mL amplified phage pool was used in rounds 2–5 of panning. In Selections 2–7, 200 µL of the 2 mL amplified phage pool was used in rounds 2–5 of panning.

4 Number of washes was increased with the number of selections performed. In all selections, there was one wash after the first round and up to either 5 or 10 washes in subsequent rounds.

5 A single wash involved filling the well with wash buffer and pipetting vigorously up and down 5 or 10 times (5X or 10X). In some of the selections, a 2-min wait between each wash was incorporated in the washing procedure as indicated by * in the table.

**Table 3:** Incidence of Pairings between the Vκ and V_H_ Clonotypes.

Light chain		kf4/Jκ5						kf4/Jκ4			kf4/Jκ2		cw9/Jκ4	ce9/Jκ1	19–15/Jκ5	TOTAL
Heavy chain	Clonotype^1^	1	2	3	4	5	6	1	2	3	1	2	1	1	1	
J558.6/DFL16.1/JH2	1				2^2^	2		2			4					10
J558.6/DFL16.1/JH3	1										1					1
J558.12/DSP2.5/JH2	1					5										5
J558.12/DSP2.5/JH2	2												18			18
J558.12/DSP2.5/JH3	1												1			1
J558.12/DSP2.5/JH3	2												1			1
J558.12/DSP2.5/JH3	3												11			11
J558.12/DSP2.5/JH3	4												5			5
J558.12/DSP2.5/JH4	1												3			3
J558.54.148/DSP2.2/JH2	1							1								1
J558.54.148/DSP2.5/JH2	1	6	1	5	16	10	2	7	3			9				59
J558.17/DSP2.5/JH2	1									1						1
J558.35/DSP2.2/JH2	1		1													1
VH7183.a30.50/DSP2.5/JH2	1							1			1					2
VH7183.a10.15/DSP2.5/JH3	1														2	2
VH7183.a15.24/DFL16.1/JH2	1													1		1
VH7183.a15.24/DSP2.3/JH4	1													2		2
VH7183.a15.24/DSP2.10/JH4	1													1		1
VH7183.a19.31/DFL16.1/JH4	1													3		3
VH7183.27b/DSP2.5/JH2	1					1										1
VHQ52.a24.72/DSP2.5/JH2	1					55					1					56
VHQ52.a27.79/DSP2.5/JH2	1		4					2								6
VH9.10/DFL16.1/JH2	1	1														1
VH9.10/DFL16.1/JH2	2	2														2
VH9.12/DFL16.1/JH2	1		1					2								3
VHVGAM3.8.a4.102/DFL16.1/JH2	1	3														3
VGK6/DSP2.3/JH2	1					1										1
TOTAL		12	7	5	18	74	2	15	3	1	7	9	39	7	2	201

1 Clonotypes were determined by IgAT analysis.

2 Numbers in each cell represent the number of times a V_L_ clonotype was found to pair with the corresponding V_H_ clonotype.

**Figure 2:**
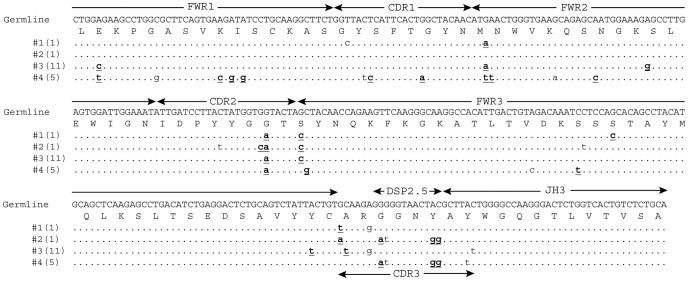
Representative IgAT analysis for V_H_ clonotypes. Four unique J558.12/DSP2.5/JH3 DNA sequences were segregated into 4 clonotypes using IgAT. Germline sequence was obtained by manually juxtaposing the available germline J558.12 sequence (GenBank: AF303843.1) with the DSP2.5/JH3 sequence derived from the first sequence in the alignment (i.e. clonotype 1).

V_H_ gene usage was less restricted than Vκ gene usage. Genes from 6 different V_H_ families that included J558 (5 genes), 7183 (5 genes), Q52 (2 genes), V_H_9 (2 genes), VHVGAM (1 gene) and VGK6 (1 gene) were utilized. The V_H_ genes recombined with 6 different D_H_ and three different J_H_ genes (Table 3). Overall, fifty-two unique VDJ sequences were identified and multiple unique sequences could be assigned to each VDJ rearrangement. For example, there were four unique J558.12/DSP2.5/JH3 sequences (Figure 2, Table 3).

Random pairing of the V-genes in a phage display library makes determining the clonality of the V-genes more difficult than for hybridoma-produced antibodies. The Immunoglobulin Analysis Tool (IgAT [25]) was used to determine if the unique sequences within specific VDJ or Vκ/Jκ recombinations likely arose from independent clones or the same clone. For example, IgAT analysis grouped the 15 unique kf4/Jκ5 sequences into 6 clonotypes (Figure 1, Table 3) and the 4 unique J558.12/DSP2.5/JH3 sequences into 4 distinct clonotypes (Figure 2, Table 3).

After all Vκ and V_H_ unique sequences were analyzed and ordered into clonotypes, the incidence of pairing between Vκ and V_H_ clonotypes was analyzed (Table 3). Overall, kf4/Jκ5 clonotypes paired with at least one heavy chain clonotype from every V_H_ family that was isolated. Other kf4 clonotypes (kf4/Jκ4 and kf4/Jκ2) also paired with clonotypes from multiple V_H_ families. None of the other light chain clonotypes (cw9, ce9, 19–15) were found paired with heavy chain clonotypes from multiple V_H_ families. For example, the cw9/Jκ4 clonotype paired with multiple J558.12 heavy chain clonotypes but did not pair with any of the other V_H_ family clonotypes. Thus, it appears that the kf4 Vκ gene was the dominant L chain used for pairing with H chains in the TT-selected library. For H chains, it appeared that there was no clear-cut dominant gene family utilized in the library, but some H chain pairings, such as J558.12/DSP2.5/JH3 and VH7183 clonotypes, were largely restricted to pairing with cw9/Jκ4 and ce9/Jκ1 clonotypes.

### Comparison Between the Anti-TT Hybridoma Library and Phage Display Library

**Figure 3:**
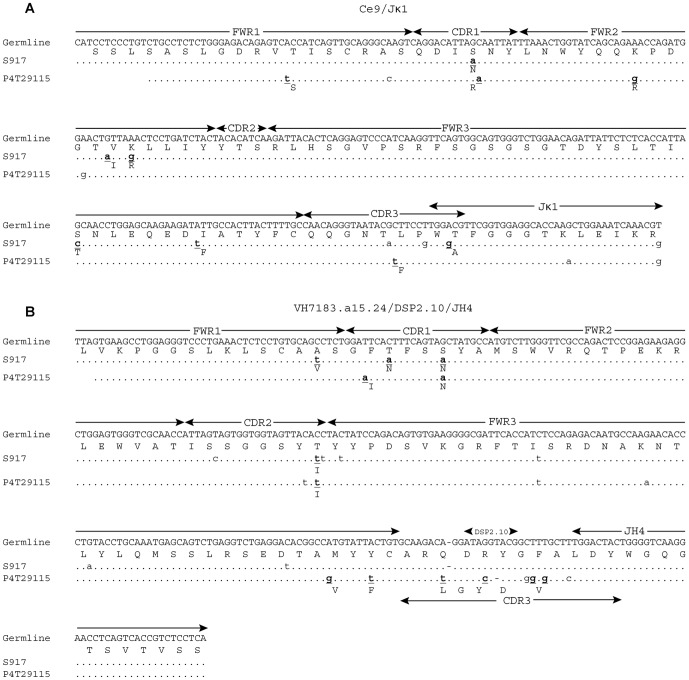
Sequence alignments of V_L_ (A) and V_H_ (B) genes from recurrent pairs between the hybridoma P4T29115 and phage display isolate S917. Replacement mutations are shown in bold font and the replaced amino acid residues are shown below each mutation. The ce9/Jκ1germline sequence was obtained by manually juxtaposing the available germline ce9 (GenBank: AJ239197.1) and Jκ1 (GenBank:V00777.1) sequences. The VH7183.a15.24/DSP2.5/JH4 germline sequence was obtained by manually juxtaposing the available germline VH7183.a15.24 sequence (GenBank:AJ851868.3) with the DSP2.5/JH4 sequence derived from S917.

**Figure 4:**
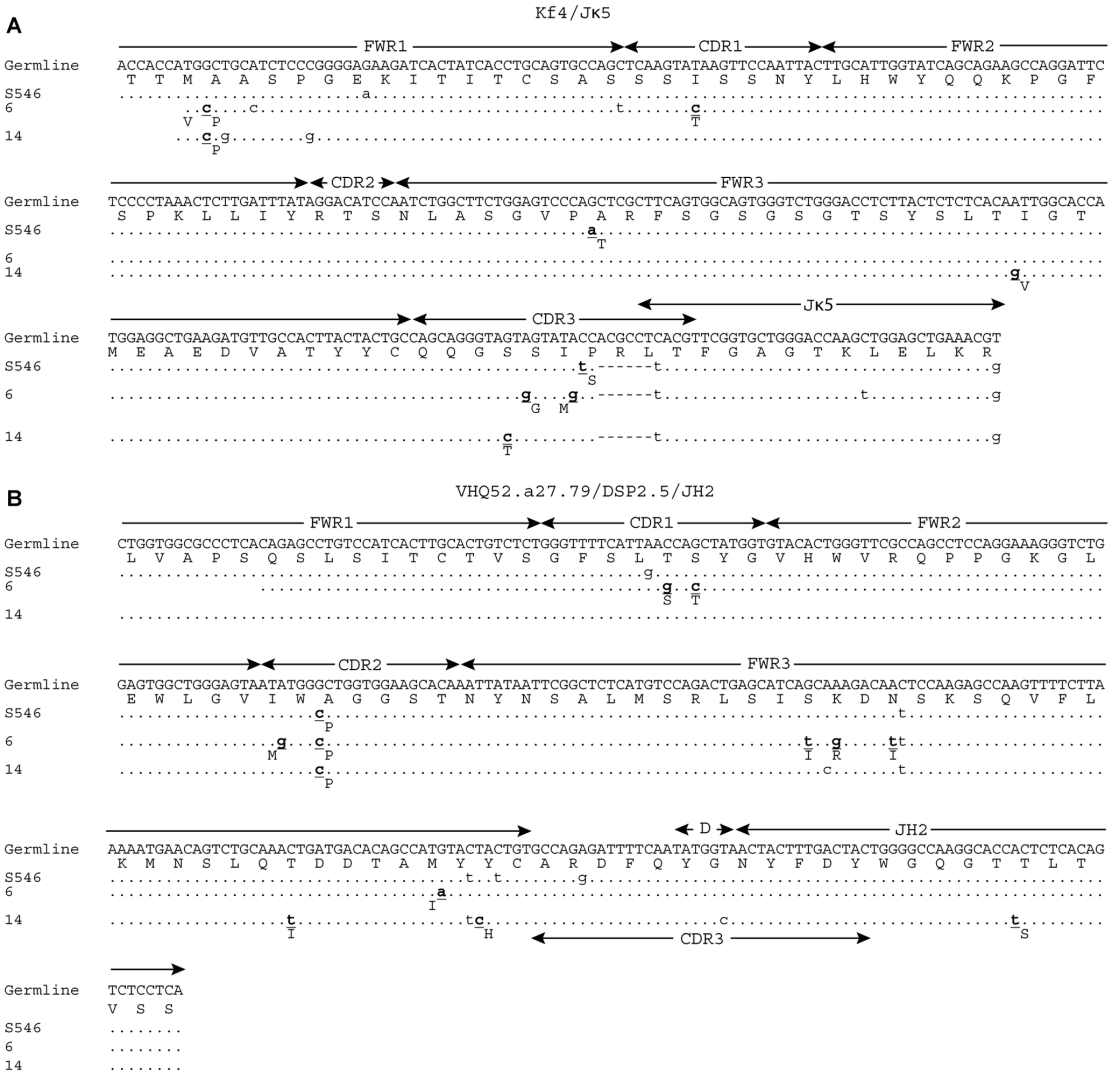
Sequence alignments of V_L_ (A) and V_H_ (B) genes from recurrent pairs between hybridomas 6 and 14 and phage display isolate S546. Replacement mutations are shown in bold font and the replaced amino acid residues are shown below each mutation. The kf4/Jκ5 germline sequence was obtained by manually juxtaposing the available germline kf4 (GenBank: AJ231229.1) and Jκ5 (GenBank:V00777.1) sequences. The VHQ52.a27.79/DSP2.5/JH2 germline sequence was obtained by manually juxtaposing the available germline VHQ52.a27.79 sequence (GenBank:AJ851868.3) with the DSP2.5/JH2 sequence derived from S546.

Comparison of the sequences between the anti-TT phage and the hybridoma antibodies revealed that there was very limited overlap between the two in both individual gene usage and V_H_-V_L_ pairs. There were only three hybridomas (6 and 14, which are clonally related, and P4T29115) that had the same V_H_-V_L_ pairs as TT-binding phage clones. Based on comparisons of the V(D)J junction in the H and L chains, phage clone S917 was not clonally related to hybridoma P4T29115 (Figure 3), however, both the L and H chain sequences in phage clone S546 are potentially clonally related to hybridoma clones 6 and 14 (Figure 4). Other than the recurrent V_H_-V_L_ pairs, only the kf4/Jκ4 light chain was utilized in both libraries, but paired with different VDJ sequences. Seven V_H_ genes were utilized in both libraries, but in different VDJ combinations, and none of the V_H_ chains in the hybridomas paired with L chains used in the phage clones.

### Binding Kinetics of Anti-TT mAbs and Fabs

In order to compare the rate and dissociation constants of the TT-specific mAbs and Fabs, the binding kinetics of all the mAbs and representative Fabs from the phage display library were determined by Surface Plasmon Resonance (SPR). When using SPR to measure the binding kinetics of an antibody-antigen interaction, immobilization of the antibody as the ligand will avoid binding avidity effects that result from the bivalency of the antibody. Immobilization of the bivalent protein enables determination of the kinetic rate constants by fitting the responses to a simple 1∶1 binding model. Comparison of antibody:antigen binding interaction to the monovalent Fab:antigen binding interaction using a similar assay format has advantages, including: higher binding activity attributed to homogeneous presentation of the ligand, availability of the binding site, and use of fresh ligand for each binding cycle.

**Figure 5:**
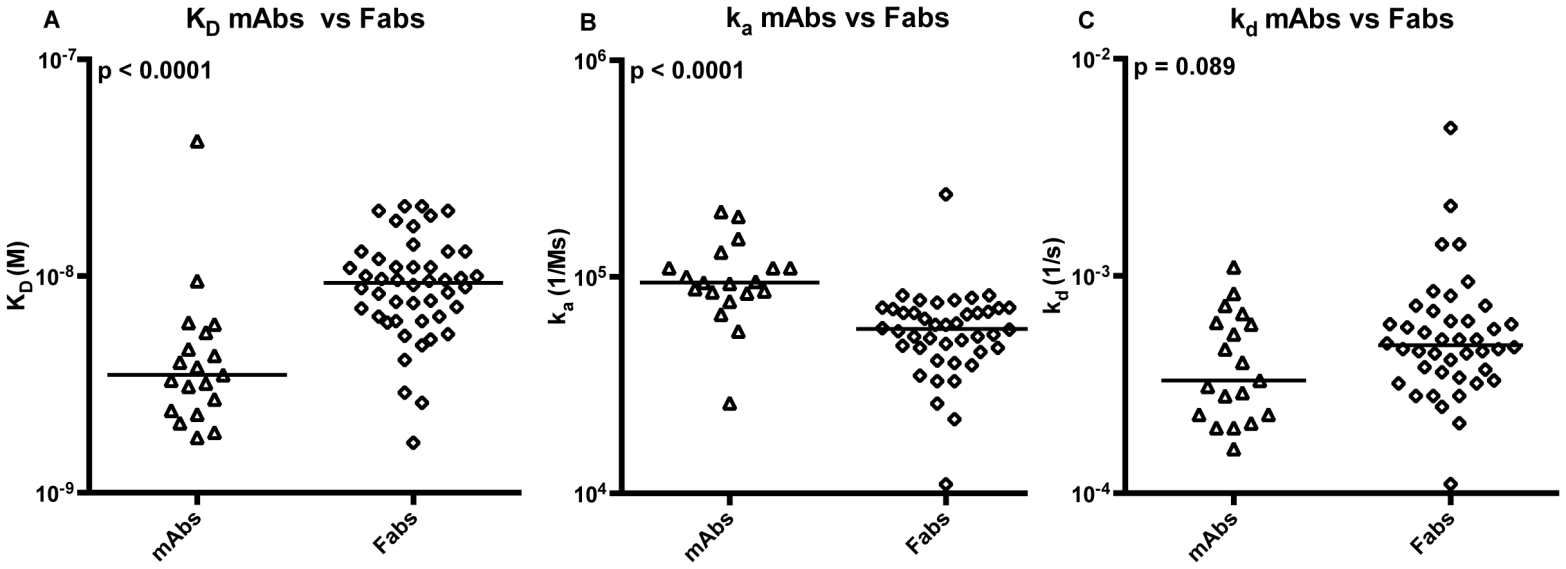
Binding Kinetics of TT-Specific mAbs from the Hybridomas and Fabs from the Phage Display Library. Different capture antibodies were used in SPR assays for mAbs (goat anti-mouse IgG1) and Fabs (goat anti-mouse IgG Fab), however, the design of the SPR assays allowed for direct comparison of the affinity values. (A) affinities (KD), (B) on-rates (ka), and (C) off-rates (kd) of TT-specific mAbs and Fabs were determined by SPR. The horizontal bars show the median values of each data set. Statistical comparisons were performed using the Mann Whitney U test.

Representative sensorgrams are shown in Figure S1. Overall, the Fabs had lower affinities than the mAbs (p<0.0001; Figure 5A, Table S2). The K_D_ values for the mAbs ranged from 1.8×10^−9^ to 4.2×10^−8^ M, with all but one antibody (P429.26) with a K_D_ in the nM range (median K_D_: 3.4×10^−9^ M) (Figure 5A, Table 1). K_D_ values for the Fabs ranged from 1.7×10^−9^ to 2.1×10^−8^ M with a median K_D_ of 9.0×10^−9^ M (Figure 5A, Table S2). Likewise, the Fab k_a_ values (range 1.1×10^4^ to 2.4×10^5^ 1/Ms) were significantly different from the k_a_ of mAb's (range 2.6×10^4^ to 2.0×10^5^ 1/Ms). The median k_a_ value for Fabs (5.7×10^4^ 1/Ms) was 1.6 fold lower than the median k_a_ value for the mAbs (9.4×10^4^ 1/Ms) (Figure 5B, Table 1 and Table S2). The k_d_ values for the mAbs and Fabs were not significantly different (range for k_d_ values of mAbs (1.6×10^−4^ to 1.1×10^−3^ 1/s) and Fabs (1.1×10^−4^ to 4.8×10^−3^ 1/s)) (Figure 5C, Table 1 and Table S2).

## Discussion

B cells from a single TT-immunized mouse were used to produce hybridomas and a phage display library in order to compare the diversity of the TT-specific immune response. We expected a greater diversity in V_H_-V_L_ pairs from the phage display library with substantial overlap between the two, because a greater number of phage clones could be screened than hybridomas generated and amplification of IgG V_H_ gene cDNA from this mouse should derive almost exclusively from TT-specific B cells. Instead, we found that the hybridoma library was more diverse in both its V_H_-V_L_ pairs and identifiable TT epitope specificity, with limited overlap in V_H_-V_L_ pairs. There was more overlap in specific V_H_ gene usage between the libraries (8/21 hybridoma and 8/16 phage clone V_H_ genes), but not the specific VDJ combinations. In addition, the hybridoma mAbs had greater affinity for TT than the phage display Fabs.

The diversity of a hybridoma library is limited by the instability of clones early in the selection process, the loss of antibody production and the ability to manage cell culture when high throughput methods are not available. Because the size and diversity of the original unselected phage display library affects the diversity and specificity of the final antigen-selected library, we maximized the size and diversity of the phage display library at every step. V-gene sequence analysis of randomly selected clones directly after cloning the Vκ and V_H_ PCR products into pCES and after expansion of the phagemid library revealed the presence of multiple Vκ and V_H_ gene families. The Vκ sequences were both germline and hypermutated and represented both high and low usage families [26]. For example, Vκ1, Vκ4/5, and Vκ19 usage is approximately 20% in adult BALB/c mice. Vκ2, Vκ12, and Vκ23 are rarely used but were detected in our expanded library.

The V_H_ genes isolated from the expanded library were from the five most utilized V_H_ families in BALB/c mice. In general, the families not detected in our library are utilized to a lesser extent in BALB/c mice and certain families; such as V_H_11 and V_H_12, are predominantly used in B1 B-cells and are rare in splenic B cells [26]–[28]. Overall the unselected and expanded libraries were genetically diverse and therefore, the limited diversity of the anti-TT Fabs is not due to a lack of diversity of the phage display library.

Different selection conditions were also used to maximize the diversity and the percentage of TT-reactive clones. While the incompatibility of some expressed sequences with phage propagation and susceptibility of others to proteolysis during propagation will lead to some loss in diversity [8], the expectation for selection of a genetically diverse pool of antigen-specific clones from the phage library was based on the multiple putative epitopes created by the structural complexity of the TT molecule.

Phage library diversity may be limited due to inefficiency of folding of the phage coat protein III (gIIIp) fused Fabs in *E.coli* and by potential toxicity of Fabs to *E. coli*. Previous reports have also highlighted the effects of varying selection parameters on the diversity and functionality of a phage library [29]–[31]. Yet, others have identified the amplification step, which is an essential step for enriching clones isolated during selection, as a co-contributor to loss of diversity in a phage display library. Considering that selection favors the best antigen binders and amplification favors the best growers, multiple rounds of selection and amplification are thought to limit the diversity of the final pool of antigen-specific clones by eliminating the majority of weak binders and slow growers [32]. We found that the majority of clones in the unselected library that contained both the heavy and light chain inserts were capable of expressing phage bearing Fab and that varying the selection conditions did not improve the genetic diversity of the antigen-specific clones isolated from our phage display library. In order to test whether repeated rounds of selection and amplification were responsible for the limited genetic diversity, we screened phage pools from earlier rounds of selection and sequenced the Vκ and V_H_ genes of the antigen-specific clones. While the percentage of antigen-specific clones in earlier phage pools was much lower than that of later pools, the V-gene diversity of such clones was very similar to that of antigen-specific clones in later rounds, suggesting that multiple rounds of selection and amplification may not be a major reason for lack of genetic diversity in our TT-specific phage clones. Even though the phage library should be enriched for TT-specific sequences, a more likely explanation for the observed limited genetic diversity may involve random pairing of Vκ and V_H_ genes, resulting in antibodies with diminished or no specificity against the target antigen. This is supported by previous reports in which construction of anti-TT libraries preserving cognate H and L chain pairing, resulted in a very diverse V-gene repertoire where a combinatorial library from the same donor was less diverse [18], [33] and in scFv phage libraries where the isolated V gene repertoire from phage were substantially different from those obtained by high throughput sequencing of V genes obtained from antigen-specific cells [10].

The V-gene repertoire of TT-specific clones isolated from our phage display library was limited to four Vκ and 16 V_H_ genes. The Vκ diversity is quite limited relative to the hybridoma panel, while the V_H_ is somewhat limited in total numbers, but half of the Vκ (2/4) and V_H_ (8/16) genes in the phage display panel were not found in the hybridoma panel. The total antigen-specific selected phage display clones contained multiple replicates/variants of some clones as well as clones that were isolated only once or a few times. This has been reported for previous phage display libraries, and suggests that isolation of low-frequency clones may require extensive screening [34].

Despite the limited V-gene diversity of TT-specific clones, multiple hypermutated variants of most V_H_ and Vκ genes were found, which is indicative of *in vivo* affinity maturation associated with a secondary immune response [16], [34], [35]. Since the naïve mouse was immunized only with TT, the mutated sequences are likely representative of the *in vivo* TT response. While some mutations were found in the framework regions, most were replacement mutations concentrated in the CDR regions, especially CDR3. The presence of different amino acid residues in CDR3 may be attributed to the mechanism of recombination that generates this region [16], [35], but others are likely a result of affinity maturation. Additionally, direct contact of either one or both CDR3 loops with antigen has been observed in most structurally characterized antigen-antibody complexes, which emphasizes the importance of diversity of the CDR3 region in antibody affinity and specificity [16].

Previous studies reported dominance of 4 V_H_ genes and V_H_ promiscuity with respect to V_L_ pairing in combinatorial libraries against TT [17], [18] and other antigens [36]. The panel of anti-TT clones from our phage display library had a moderate level of V_H_ gene promiscuity, but a high level of Vκ gene promiscuity. In fact, the Vκ gene kf4 is the most promiscuous gene in our library, as it was found in 76% of the isolated antigen-specific clones and was able to pair with at least one V_H_ gene from each V_H_ family isolated. This suggests that in this mouse, the kf4 gene, which was also used in two of the independent hybridomas, plays an important role in binding to TT. Our results suggest that the specificity of the isolated antibodies was a result of both Vκ and V_H_ genes.

The median affinities of both mAbs and Fabs were in the nM range but, the affinities of the Fabs were, on average, lower than the affinities of mAbs and, with the exception of one mAb (P429.26), were spread over a wider range. The presence of anti-TT Fabs with slightly lower affinities (10^−8^ M) may be due to utilization of non-cognate Vκ/V_H_ pairs, which is similar to the comparison between human anti-TT Fab cognate pairs and combinatorial pairs [18]. Considering that a small k_a_ and/or a large k_d_ often lead to low affinity constants (*i.e*., strong affinity binding), whereas a large k_a_ and/or a small k_d_ lead to a high affinity constants (*i.e*., weak affinity binding) [33], the slightly lower median k_a_ coupled with the slightly higher median k_d_ of Fabs explain the overall lower Fab K_D_ values.

The observed micro-heterogeneity in the V-gene sequences of the TT-reactive clones also agrees with previous reports suggesting that the improvement in affinity is more likely the result of many small additive changes rather than a few large effects [16]. The lower affinity observed for TT-specific clones from the phage display library relative to the hybridoma library highlights the fact that although binding properties of an antibody isolated from a phage display library may not be ideal for therapeutic purposes, directed evolution can be used to improve the binding properties of a such antibody towards a specific antigen [8].

## Materials and Methods

### Anti-TT mAbs

A 6-week-old female BALB/c mouse was immunized intraperitoneally (IP) with 100 µg of TT (Calbiochem-EMD Millipore) emulsified in 100 µL of complete Freund's adjuvant and boosted IP 21 days later.

The spleen was harvested seven days after the boost and mashed into suspension in a Petri dish containing 20 mL of RDG media (50% RPMI, 50% DMEM, 10% FBS, 20 ug/ml Gentamicin, 2-Mercaptoethanol). Cells were strained, washed in RDG media and finally suspended in 20 mL of RDG media. Half of the spleen cell suspension was used for fusion to SP 2/0 cells. The SP2/0 and spleen cells were pooled at a SP2/0: spleen ratio of 1∶2 and centrifuged at 200×g for 10 min. The cell pellet was suspended in 1 mL of warm PEG 1500 (Roche) and the tube was incubated at 37°C for 1 min. Four mL of RDG were added, and the tube was incubated at 37°C for 1 min. The last two steps of this process were repeated with a two-fold increase in the volume of RDG, and the cells were centrifuged at 100×g for 10 min. The final cell pellet was suspended in 175 mL of RDG +10% FBS + HAT (Sigma), and a series of two-fold dilutions of the fused cells were plated in 96-well plates. Supernatants from fusion wells were screened by ELISA for positive binding to TT and negative binding to BSA as a control protein antigen. For ELISA, Microfluor microtiter plate (Dynex Technologies) wells were coated with 0.5 µg/well of TT or BSA in 100 µL of coating buffer (0.1 M sodium bicarbonate, pH 8.6) at 4°C overnight. Wells were rinsed 3x with 300 µL of PBS/0.5% BSA and blocked at room temperature (RT) for 2 hours with a final 300 µL of PBS/0.5% BSA. After removal of the blocking buffer, 100 µL of supernatant from fusion wells were added to wells and the plate was incubated at RT for 1 hour. Plates were washed 4x with PBS/0.1% Tween (PBST), and 100 µL/well of alkaline phosphatase-labeled goat anti-mouse heavy and light chain antibody (Southern Biotechnology Associates) were added to the plate. After incubating the plate at RT for 1 hour, the wells were washed 4x with PBST, and 100 µL of 5 µg/mL 4-methylumbellyferyl phosphate were added to the wells. Plates were read in a microplate fluorimeter. Cells from positive wells were cloned by limiting dilution and rescreened as described above. Supernatants from TT-positive clones were screened for binding to two preparations of recombinant fragment C (H_C_) (prepared by Dr. Willie Vann, CBER or purchased from Roche) or tetanus L chain (List Biological Laboratories) by ELISA. ELISAs were performed as described above except that wells were coated with 100 µL of 1 µg/mL of H_C_ in coating buffer at 4°C overnight and a goat anti-mouse IgG1-HRP antibody was use for detection. Antibodies were isotyped by ELISA with a mAb-based isotyping kit (Pharmingen) according to the manufacturer's instructions.

RNA from TT hybridomas was obtained using the TRIZOL method (Invitrogen). cDNA was synthesized from total RNA using the SuperScript III First-Strand Synthesis System for RT-PCR (Invitrogen) and an oligo(dT) primer. Vκ and V_H_ sequences were amplified by PCR using the same Vκ and V_H_ primary primers (Table S3) used for constructing the phage library [22], [37] and the Roche FastStart High Fidelity PCR System (Roche Applied Science). Each 50 µL reaction contained 100 ng of template, 5 µL of 10X Buffer with 18 mM MgCl_2_, 10 nmol of dNTP mix, 2.5 U of FastStart enzyme, and 20 pmol each of the appropriate forward and reverse primers. Cycling conditions consisted of 95°C for 5 min followed by 30 cycles of 95°C for 1 min, 50°C–60°C for 1 min, and 72°C for 1 min, followed by a 7-min extension at 72°C. PCR products were ligated into the TOPO vector (Invitrogen) and sequencing of purified plasmids was performed by Eurofins MWG Operon. Vκ and V_H_ sequences are deposited in Genbank (accession numbers KJ398432-KJ398471 and KJ398474-KJ398475).

### Ethics Statement

Mice were maintained in accordance with CBER's Institutional Animal Care and Use Committee regulations according to protocol WO-2007-60.

### Phage Library Construction

RNA was isolated from half of the harvested spleen cell suspension using the TRIzol method (Invitrogen). First-strand cDNA was synthesized as described above and Vκ and V_H_ sequences were amplified by PCR using the Roche FastStart High Fidelity PCR System. Each 50 µL reaction contained 100 ng of template, 5 µL of 10X Buffer with 18 mM MgCl_2_, 10 nmol of dNTP mix, 2.5 U of FastStart enzyme, and 20 pmol each of the appropriate forward and reverse primers. For both Vκ and V_H_ PCRs, the annealing temperature was set low enough to maximize the amplification of all Vκ and V_H_ genes. Vκ and V_H_ sequences were amplified in a single PCR and nested PCR, respectively using the primers listed in Table S3. The reverse primer used for amplifying V_H_ sequences was specific for IgG. The Vκ and primary V_H_ PCR conditions were 95°C for 5 min followed by 30 cycles of 95°C for 1 min, 50°C for 1 min, and 72°C for 1 min, followed by a 7-min extension at 72°C. For the V_H_ nested PCR, the annealing temperature was raised to 60°C and the annealing time was increased to 1.5 min. Vκ and V_H_ PCR products were pooled separately and purified using the MinElute PCR Purification Kit (Qiagen).

### Cloning of Vκ and V_H_ Genes into the pCES Phage Display Vector

Purified Vκ and V_H_ PCR products were sequentially cloned into the pCES phage display vector, which is designed to express Fabs [38]. First, 5 µg of Vκ PCR products and 10 µg of vector DNA were digested with 10 U/µg ApaLI and AscI (New England BioLabs) at 37°C overnight. Digestion of pCES with ApaLI and AscI removes the human Cκ insert and allows cloning of the full murine VκCκ PCR product. The hybridoma sequences do not contain these restriction sites and therefore would not be eliminated during the cloning process. Digests were gel-purified using the MinElute Gel Extraction kit (Qiagen) and ligations were performed overnight at RT with 350 ng PCR products, 1 µg vector and 30 units of T4 DNA ligase (Invitrogen). Ligations were ethanol-precipitated and resuspended in 15 µL of water. Transformations were performed at 2.5 kV, 25 µF, and 200 Ω in a BioRad Gene Pulser using 300 µL of electrocompetent XL1-Blue *E. coli* (Stratagene) and 15 µL of ligation product in a 2 mm cuvette. After Vκ cloning, thirty colonies from titer plates were picked for sequencing to assess the diversity of the Vκ genes cloned into the vector. The entirety of the remaining electroporated cells (10^7^–10^8^ transformants) were spread onto 150-mm LB/carb plates and incubated at 37°C overnight. Colonies were scraped off the 150-mm plates into 10 mL of LB media and plasmid DNA was isolated with the HiSpeed Plasmid Purification Kit (Qiagen). Plasmid DNA served as the vector for cloning of the V_H_ PCR products. Five µg of V_H_ PCR products and 10 µg of vector were digested with 10 U/µg of SfiI (New England Biolabs) for 5 hours at 50°C and then with 10 U/µg of NotI-HF (New England Biolabs) at 37°C overnight. Digestion of the vector with SfiI and NotI removes the human CH1 domain from the vector. Digest purification, ligation, electroporation and titrations were performed as described above for Vκ. After transformation, the size of the final library was 1.3×10^7^ clones. Colonies from titration plates were picked for sequencing to assess the diversity of the V_H_ and Vκ genes cloned into the vector. All of the library was plated on 150 mm LB/carb plates and after overnight growth, the final V_H_/Vκ transformant library was scraped off and suspended in 10 mL of LB and stored as glycerol stocks.

### Confirmation of Fab expression

Fifty µL of the V_H_/Vκ library glycerol stock was inoculated into 10 mL of 2YTA (1.6% tryptone, 1% yeast extract, 0.5% NaCl, 1% glucose, 100 µg/mL carbenicillin) media and incubated at 37°C and 200 rpm until the OD_600_ reached 0.4 to 0.5. Phage were rescued with VCS-M13 helper phage (gift of Dr. Christoph Rader, NIH) as previously described [39]. Rescued phage particles in the supernatant were precipitated by addition of 1/5 volume of 20% PEG in 2.5 M NaCl and incubation on ice for ≥30 min. The precipitated phage pellet was collected by centrifugation at 4000×g at 4°C for 40 minutes and resuspended in 1 mL of 1X PBS. Ninety µL aliquots of SS320 *E. coli* cells in the exponential growth phase were infected with 10 µL of serial dilutions of the phage. The phage-infected SS320 cells were incubated without shaking at 37°C for 30 minutes and spread onto YTAG plates. After incubation of the plates at 37°C overnight, 376 colonies were picked to wells of a 96-well culture plate containing 150 µL 2YTA/1% glucose and grown for 3 hours at 37°C, 250 rpm. For isolation of phagemid DNA, deep well culture plates containing 750 µL of 2YTA/1% glucose were inoculated with 5 µL of bacteria from the 3 hour cultures. To induce Fab expression, 1 µL of 100 mM IPTG was added to each well of the 3 hour culture plates. The deep well plates and the IPTG induction plates were grown overnight at 37°C and 27°C, 250 rpm, respectively.

ELISA plates (Nunc Maxisorp) were coated overnight at 4°C with 100 µL of 1 µg/mL goat anti-mouse light chain (Jackson). Plates were blocked with PBS/0.1% BSA for two hours at room temperature and washed once with PBS. One hundred microliters of supernatants from overnight IPTG induction cultures were added to ELISA wells and incubated for 1 hour at room temperature. After washing 3X with PBS/0.1% tween 20, wells received 100 µL of HRP-labeled goat anti-mouse Fab (Sigma) and plates were incubated for one hour at room temperature. Plates were washed 3X with PBS-tween and developed with SureBlue TMB Microwell Peroxidase Substrate (KPL). Plates were incubated in the dark at RT for 10 minutes, then 100 µL of 1 N HCl were added to each well to stop the reactions and plates were read at 450 nm in an ELISA reader. Plasmid DNA was isolated from deep well cultures using the Zyppy plasmid miniprep kit (Zymo research). Plasmids were assessed for the presence of V_H_ and V_L_ by DNA sequencing.

In a second experiment, phage were rescued at small scale from 172 individual colonies of the XL-1 blue glycerol stock library and assessed for Fab expression by ELISA as described above. The glycerol stock library was plated for colony isolation and individual colonies were inoculated into wells of a deep well culture plate containing 500 µL 2YT/ampicillin and 1% glucose. After overnight incubation at 37°C with shaking at 300 rpm, 5 µL of each overnight culture were used to inoculate a second deep well culture plate containing 500 µL of 2YTA/1% glucose per well. Plates were incubated for 3 hours at 37°C with shaking at 300 rpm. Helper phage (1×10^10^ pfu) was added to each well and plates were incubated for 30 min at 37°C without shaking, followed by 1 hour at 37°C with shaking. Bacteria were pelleted by centrifugation and media was discarded. Bacteria were resuspended in 500 µL 2YT containing ampicillin (100 µg/mL), kanamycin (25 µg/ml) and tetracycline (50 µg/ml) and incubated overnight at 37°C with shaking at 300 rpm. Each supernatant from the overnight cultures was tested in the ELISA described above for Fab expression on the phage. Plasmid DNA from individual bacterial pellets was isolated using the Wizard miniprep kit (Promega) and digested with ApaL1 and Asc1 to determine the presence of LC and HC PCR products in the pCES vector.

### Antigen-Specific Phage Selection by Panning

In order to obtain live phage particles for use in selection, 10-50 µL of the V_H_/Vκ library glycerol stock was inoculated into 10 mL of 2YTA (1.6% tryptone, 1% yeast extract, 0.5% NaCl, 1% glucose, 100 µg/mL carbenicillin) media and incubated at 37°C and 200 rpm until the OD_600_ reached 0.4 to 0.5. This culture will be referred to as the expanded library hereafter. Twenty random colonies from the expanded library were sequenced to assess the diversity of the library. Phage from the expanded library were rescued with VCS-M13 helper phage and purified as described above.

The resulting phage library (2.1×10^13^ pfu/mL) was subjected to five rounds of selections at 37°C. Details for each selection are described in Table 2 and were adapted from previously published methods [39], [40]. Selection parameters were varied in order to maximize the number and diversity of TT reactive clones (Table 2). Selections of the phage (inputs) were performed on ELISA plate wells (Nunc Maxisorp) coated with TT, H_C_ (List Biological Laboratories), or tetanus toxin light chain (TT-LC, List Biological Laboratories). ELISA plate wells were coated overnight at 4°C with antigen in 50 µL of coating buffer. Antigen-coated wells were blocked with 150 µL/well of 3% (w/v) BSA in TBS at 37°C for 1 hour. Blocking solution was removed and 10-100% of input phage was added and incubated at 37°C for 15–120 min (Table 2). After washing, bound phage was eluted with 50 µL/well of 100 mM triethylamine (Acros Organics) at RT for 5 min. Eluted phage (output) was neutralized with 0.5 volume of 1 M Tris-Cl, pH 7.4 and amplified in exponentially growing XL1-Blue *E. coli* cells (OD_600_∼0.7) as previously described [39], [40]. The phage titer in each input and output phage pool was determined by infecting 90 µL aliquots of XL1-Blue *E. coli* cells in the exponential growth phase with 10 µL of serial dilutions of the phage pools. The phage-infected XL1-Blue cells were incubated without shaking at 37°C for 30 minutes and spread onto YTAG plates. After incubation of the plates at 37°C overnight, colonies were counted and titers were calculated.

The success of the selection process was monitored by testing the input and output phage pools in an ELISA. The wells of an ELISA plate (Nunc Maxisorp) were coated with 0.05 µg of antigen (TT, H_C_, or TT-LC) in 50 µL of coating buffer at 4°C overnight. The antigen-coated wells were blocked with 150 µL of blocking buffer at 37°C for 1 hour. Blocking solution was removed from the wells, 50 µL/well of serially diluted phage pools were added, and the plate was incubated at 37°C for 2 hours. Plates were washed three times with PBST and phage bound to TT were detected with 50 µL/well HRP-conjugated anti-M13 antibody (GE Healthcare) and/or HRP-conjugated goat anti-mouse kappa antibody (Southern Biotechnology Associates) and SureBlue TMB Microwell Peroxidase Substrate (KPL). The plate was incubated in the dark at RT for 30 min–1 hour, then 100 µL of 1 N HCl were added to each well to stop the reactions and plates were read at 450 nm in an ELISA reader.

### Screening for Individual Antigen-Specific Clones

After five rounds of panning, the final output phage pool was screened for single antigen-specific clones as described [39]. Briefly, an exponentially growing culture of SS320 cells (Lucigen), a non-suppressor strain of *E. coli*, was infected with approximately 10^3^ pfu of the 5^th^ round output, plated onto YTAG plates, and incubated at 37°C overnight. Resulting colonies were inoculated into 150 µL of 2YT broth supplemented with 1% glucose and 100 µg/mL carbenicillin in 96-well plates (Corning Costar) and incubated with shaking at 30°C for 3 hours. Expression of Fab fragments was induced by addition of 1.5 µL/well of 100 mM IPTG (Fermentas). Culture supernatants containing the soluble Fab fragments were tested for binding to TT, H_C_, or TT-LC in an ELISA (described above) with HRP-conjugated goat anti-mouse kappa antibody (Southern Biotechnology Associates) serving as the secondary antibody. Positive (*i.e*. antigen-specific) clones were defined as those resulting in an OD_450_ reading three times above the background (1% BSA in 1X TBS). Plasmid DNA was isolated from the positive clones and digested with the appropriate enzymes to confirm the presence of the Vκ and V_H_ inserts. Each clone was sequenced to determine the Vκ and V_H_ diversity of the antigen-selected phage display library. Vκ and V_H_ sequences are deposited in Genbank (accession numbers KJ415588-KJ415677).

Vκ and V_H_ families that were not detected in the selected library were confirmed to be present in the unselected library by PCR that employed gene-specific primers (Table S1) based on germline V-gene sequences obtained from IgBLAST [23] or IMGT [24]. Template DNA was obtained by heating 10 µL of the unselected TT library XL1-Blue glycerol stock diluted in 500 µL of water to 100°C for 10 min, centrifuging the tube, and transferring the supernatant into a clean tube. Each PCR contained 1 µL of this supernatant as template. PCR products were TOPO cloned into the pCR4-TOPOR vector (Invitrogen) and sequenced.

### Large Scale Fab Expression

Fab expression was induced in large culture volumes (100 mL) to produce a sufficient amount of each Fab for Surface Plasmon Resonance (SPR) analysis. Ten picograms of plasmid DNA from TT-specific phage clones were transformed into 100 µL of chemically competent SS320 *E. coli* cells (Lucigen) and grown at 37°C and 250 rpm for 1 hour. Fifty µL of each transformation was spread onto a YTAG plate, and plates were incubated at 37°C overnight. Single bacterial colonies were grown in 100 mL of 2YT media supplemented with 100 µg/mL carbenicillin and 0.1% glucose at 30°C and 250 rpm until OD_600_ reached 1.2, then IPTG was added to a final concentration of 0.1 mM, and the culture was incubated at 25°C and 250 rpm overnight. After pelleting the bacteria by centrifugation, the periplasmic fraction was extracted by osmotic shock consisting of resuspending the pellet in 1 mL of ice-cold PBS containing 0.94 M NaCl and incubation on ice for 30 minutes [18]. Bacteria were centrifuged at 14,000×g and 4°C for 20 minutes, and supernatant was filtered through a 0.22 µm filter and stored at −20°C.

### Analysis of Hybridoma and Phage Display Sequences for Clonality

The hybridoma and phage display V_H_ and V_L_ were analyzed with the immunoglobulin analysis tool (IgAT). IgAT is a Microsoft Excel based software that applies certain criteria to output files from IMGT-HighV Quest to determine clonality. Clonality is confirmed if the same V(D)J genes are used, CDR3 lengths are identical, and the CDR3 region is highly homogenous, with 10% or less difference in nucleotide sequences. Based on these clonality criteria, IgAT assigns the unique sequences within a particular Vκ/Jκ or VDJ rearrangement into potentially clonally related sequences that are considered clonotypes.

### SPR Analysis

Affinities of all TT-reactive hybridoma mAbs (Table 1) and representative Fabs (one from each clonotype) from the phage display library (Table 3) were determined by SPR on a Biacore T200 instrument (GE Healthcare). Goat anti-mouse IgG1 antibody (Bethyl Laboratories) and a goat anti-mouse IgG Fab Specific antibody (Sigma) were immobilized on a CM5 biosensor by standard amine coupling. Supernatants from hybridoma cell cultures or Fabs from the periplasmic extract were diluted in 1X HBST (150 mM NaCl, 10 mM HEPES, 0.05% Tween 20, pH 7.5). The mAbs and Fabs were captured by the immobilized goat anti-mouse IgG1 and goat anti-mouse IgG Fab Specific antibody, respectively. Concentrations of TT ranging from 0 to 180 nM in 1X HBST were run over the sensor at 20 µL/min for 4 minutes. Dissociation was followed for 10 minutes, and the surface was regenerated with 10 mM glycine at pH 2.0. The Biacore T200 evaluation software was used to fit the data to a 1∶1 or a two-state interaction model (in a few cases where the 1∶1 fit was poor) and calculate the rate (k_a_ and k_d_) and dissociation (K_D_) constants. The validity of using the two platforms was verified by cloning anti-TT mAb V_H_ and V_L_ sequences into pCES and comparing the kinetics of the *E. coli* expressed Fab to the kinetics of the mAb (data not shown).

## Supporting Information

Figure S1 **Representative SPR sensorgrams for monoclonal antibody and Fab TT kinetic assays.**
(PDF)

Table S1 **Vκ and V_H_ Gene-Specific Primers.**
(DOC)

Table S2 **Binding Kinetics of Representative TT-specific Fabs from the Phage Display Library.**
(DOC)

Table S3 **PCR Primers for the Amplification of Mouse Vκ and VH Gene Repertoires for Phage Display Library.**
(DOC)
