# Supplementary figures and images for: Diversity of the Antibody Response to Tetanus Toxoid: Comparison of Hybridoma Library to Phage Display Library
Source: PLoS One. 2014 Sep 30;9(9):e106699. doi: 10.1371/journal.pone.0106699 (PMC4182348; doi:10.1371/journal.pone.0106699)

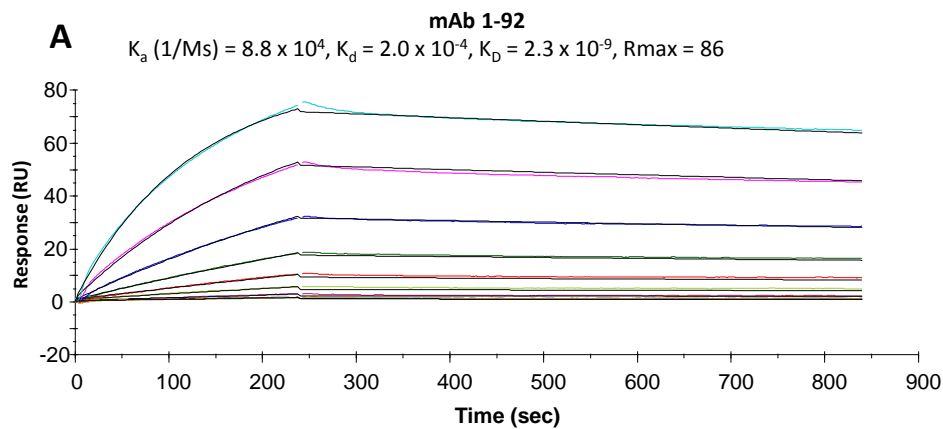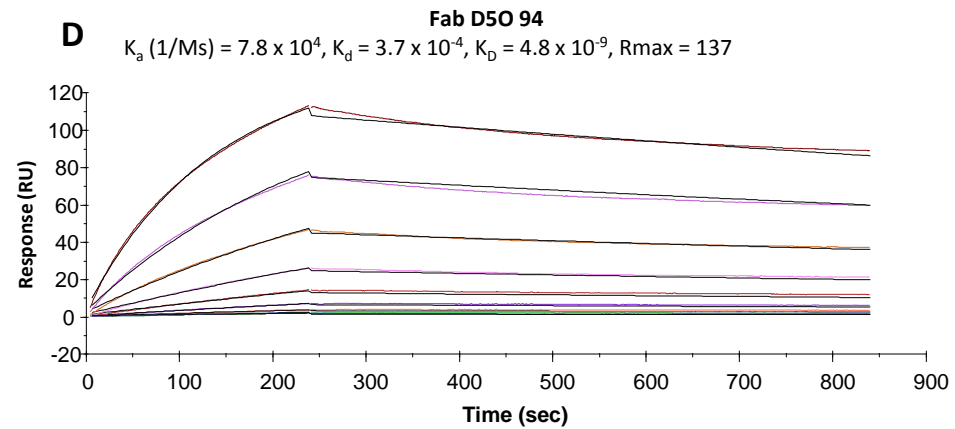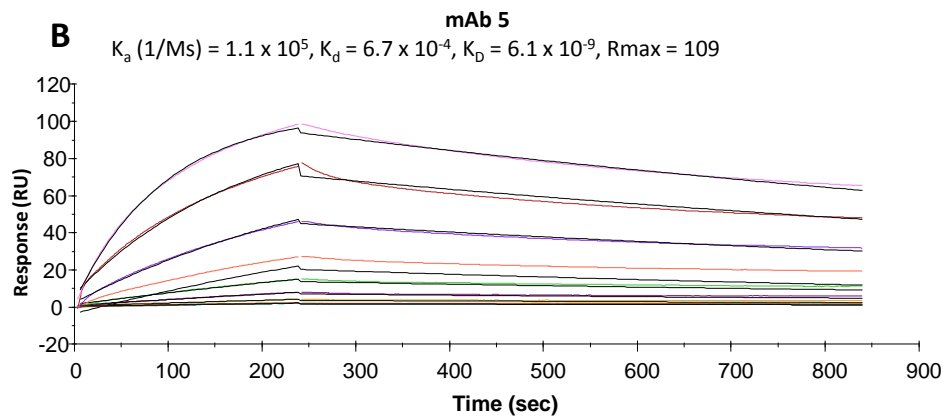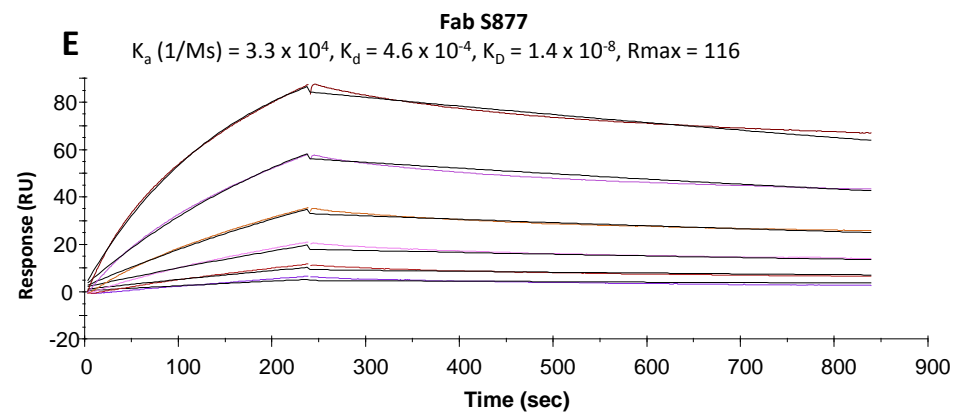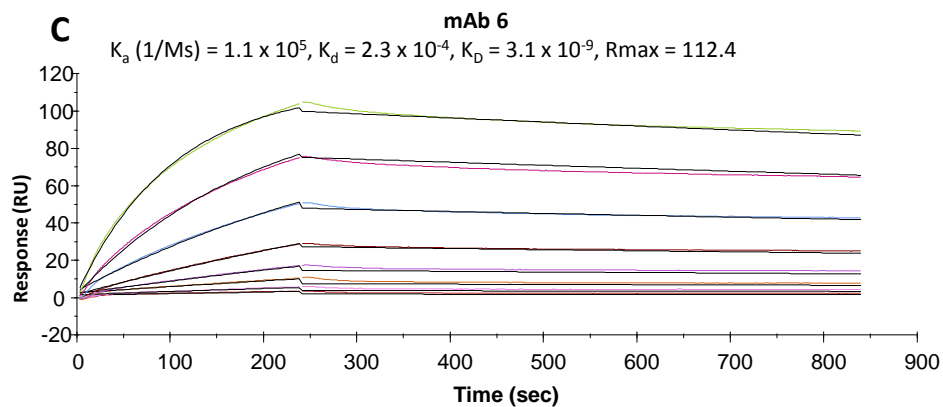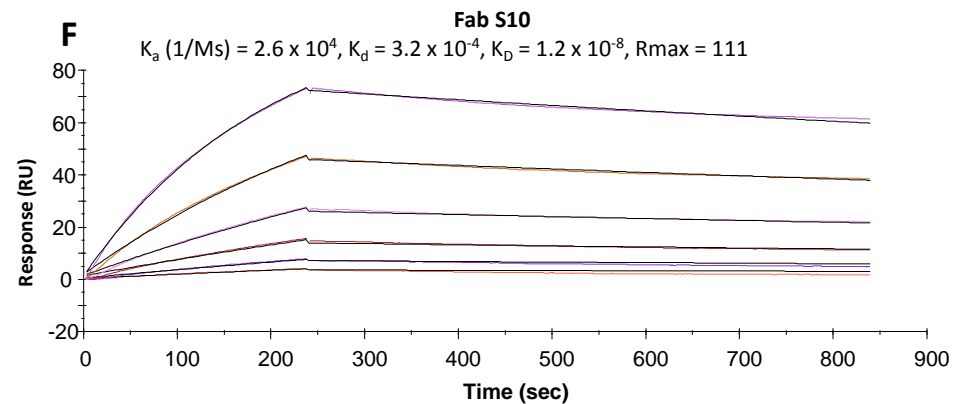

Supplement: Figure S1 — Representative SPR sensorgrams for monoclonal antibody and Fab TT kinetic assays. (PDF) [file pone.0106699.s001.pdf]
