# Supplementary material for: Diversity of the Antibody Response to Tetanus Toxoid: Comparison of Hybridoma Library to Phage Display Library
Source: PLoS One. 2014 Sep 30;9(9):e106699. doi: 10.1371/journal.pone.0106699 (PMC4182348; doi:10.1371/journal.pone.0106699)
Supplement: Table S1 — Vκ and VH Gene-Specific Primers. (DOC) [file pone.0106699.s002.doc]

Supplementary Table 1. Vκ and V_H_ Gene-Specific Primers

| **Vκ Primers (5’-3’)** | |
| --- | --- |
| bv9F | TGGTAGTAGCTTAAACTGGCTTCAG |
| bv9R | AGAATCTAAACTGGATGTGGCGTAG |
| 8-24F | GAGCCTTTTAAATAGTAGCAATCAA |
| 8-24R | CCTAGTGGATGCAAAGTATACCAGA |
| 23-48F | ACCAGTGTATGCTTGTGCCAATGC |
| 23-48R | AGTCTCCAGCCATCCTGTCTGTGA |
| 22-33F | CCATTAGTTGCACGGCCAGTGAGAG |
| 22-33R | AACCTGATCACTGCTGATGGTCAGA |
| by20F | GCTATAGGAGAAAAAGTCACCATCA |
| by20R | AATCTGCAACATCTTCTGAGAGCAT |
| gj38cF | AGGCAAGCCAAGACATTAACAAGTA |
| gj38cR | ACTGTAGACAATAATAAGTTGCAAT |
| gm33F | GACAGAGTCACCATTACTTGCAAGG |
| gm33R | GTCTGAAGACTGGTAATGCTGAGAG |
| gr32F | TTACCATCACTTGCCATGCCAGTCA |
| gr32R | AGTGGCAATGTCTTCAGGCTGCAGG |
| if11F | CAGGGCACTAGCATTAATTTAAACT |
| if11R | GCTGCTGATGGTGAGAGTGAAATCT |
| rf104F | GCATTAGCAAATATTTAGCCTGGTA |
| rf104R | ACGGGTATTCATTATGCTGTTGACA |
| Vκ10F | TCCTCCCTGTCTGCCTCTCTGGG |
| Vκ10R | AGACCCACTGCCACTGAACCTTGATG |
| **V_H_ Primers (5’-3’)** | |
| VHX24.a1.84F | GTAGATACTGGATGAGTTGGGTCCG |
| VHX24.a1.84R | AAGGGCTGTGTCCTCAGATCTCACT |
| VH22.1F | GGATTCACTTTCAGTAACTACTGGA |
| VH22.1R | GTGTCTTCAGCCCTTAAGTTGTTCA |
| V13F | GGATTCACCTTCACTGATTACTACA |
| V13R | CCTCAGCTCTCAGGGTGTTCATTTG |
| VH3609F | GGCCTGTACTTTCTCTGGGATTTCA |
| VH3609R | GGAATACTTGGTTTGTTGGAGGTCTC |
| Vh10.2aF | AGCCTAAAGGGTCATTGAAACTCTC |
| Vh10.2aR | CAGATAGAGCATGCTTTGTGAATCA |
| VH11.1.48F | GACTCTCTTGTGAAGGCTCAGGGTT |
| VH11.1.48R | ATTGCTCATCTGCAGGTACAGGGTG |
| SM7.1.44F | AAGACTACTATATGCACTGGGTGAA |
| SM7.1.44R | ACAGTAATAGACGGCAGTGTCCTCA |
