# Supplementary material for: Diversity of the Antibody Response to Tetanus Toxoid: Comparison of Hybridoma Library to Phage Display Library
Source: PLoS One. 2014 Sep 30;9(9):e106699. doi: 10.1371/journal.pone.0106699 (PMC4182348; doi:10.1371/journal.pone.0106699)
Supplement: Table S2 — Binding Kinetics of Representative TT-specific Fabs from the Phage Display Library. (DOC) [file pone.0106699.s003.doc]

Supplementary Table 2. Binding Kinetics of Representative TT-specific Fabs from the Phage Display Library

| **Clone** | **V**κ/Jκ | **V_H_/D_H_/J_H_** | **K_a_**  **(1/Ms)** | **K_d_**  **(1/s)** | **K_D_**  **(M)** | **Fab capture** | **Rmax** |
| --- | --- | --- | --- | --- | --- | --- | --- |
| D5O 94 | kf4/Jκ5 (4)^1^ | J558.6/DFL16.1/JH2 | 7.8 x 10^4^ | 3.7 x 10^-4^ | 4.8 x 10^-9^ | 330 | 137 |
| S934 | kf4/Jκ5 (5) | J558.6/DFL16.1/JH2 | 4.0 x 10^4^ | 5.1 x 10^-4^ | 1.3 x 10^-8^ | 410 | 133 |
| S1117 | kf4/Jκ5 (5) | J558.12/DSP2.5 /JH2 (1) | 5.7 x 10^4^ | 5.5 x 10^-4^ | 9.6 x 10^-9^ | 480 | 126 |
| D5O 50 | kf4/Jκ5 (1) | J558.54.148/DSP2.5/JH2 | 7.2 x 10^4^ | 2.1 x 10^-4^ | 2.9 x 10^-9^ | 448 | 146 |
| S1072 | kf4/Jκ5 (2) | J558.54.148/DSP2.5/JH2 | 3.9 x 10^4^ | 2.5 x 10^-4^ | 6.5 x 10^-9^ | 330 | 123 |
| S572 | kf4/Jκ5 (3) | J558.54.148/DSP2.5/JH2 | 4.9 x 10^4^ | 3.2 x 10^-4^ | 6.5 x 10^-9^ | 415 | 116 |
| S827 | kf4/Jκ5 (4) | J558.54.148/DSP2.5/JH2 | 6.9 x 10^4^ | 2.8 x 10^-4^ | 4.1 x 10^-9^ | 247 | 65 |
| S807 | kf4/Jκ5 (5) | J558.54.148/DSP2.5/JH2 | 4.1 x 10^4^ | 4.4 x 10^-4^ | 1.1 x 10^-8^ | 476 | 109 |
| S1054 | kf4/Jκ5 (6) | J558.54.148/DSP2.5/JH2 | 4.7 x 10^4^ | 3.6 x 10^-4^ | 7.7 x 10^-9^ | 403 | 116 |
| S877 | kf4/Jκ5 (2) | J558.35/DSP2.2/JH2 | 3.3 x 10^4^ | 4.6 x 10^-4^ | 1.4 x 10^-8^ | 444 | 116 |
| D5O 47 | kf4/Jκ5 (5) | VH7183.27b/DSP2.5/JH2 | 4.7 x 10^4^ | 8.1 x 10^-4^ | 1.7 x 10^-8^ | 490 | 71 |
| S10 | kf4/Jκ5 (5) | VHQ52.a24.72/DSP2.5/JH2 | 2.6 x 10^4^ | 3.2 x 10^-4^ | 1.2 x 10^-8^ | 285 | 111 |
| S546 | kf4/Jκ5 (2) | VHQ52.a27.79/DSP2.5/JH2 | 6.7 x 10^4^ | 4.1 x 10^-4^ | 6.1 x 10^-9^ | 460 | 118 |
| S891 | kf4/Jκ5 (1) | VH9.10/DFL16.1/JH2 (1) | 5.6 x 10^4^ | 5.1 x 10^-4^ | 9.1 x 10^-9^ | 400 | 88 |
| S1052 | kf4/Jκ5 (1) | VH9.10/DFL16.1/JH2 (2) | 2.2 x 10^4^ | 4.7 x 10^-4^ | 2.1 x 10^-8^ | 464 | 68 |
| D5O 127 | kf4/Jκ5 (2) | VH9.12/DFL16.1/JH2 | 5.3 x 10^4^ | 2.8 x 10^-4^ | 5.3 x 10^-9^ | 500 | 65 |
| S1034 | kf4/Jκ5 (1) | VHVGAM3.8.a4.102/DFL16.1/JH2 | 3.3 x 10^4^ | 6.9 x 10^-4^ | 2.1 x 10^-8^ | 445 | 75 |
| S147 | kf4/Jκ5 (5) | VGK6/DSP2.3/JH3 | 5.4 x 10^4^ | 6.2 x 10^-4^ | 1.1 x 10^-8^ | 624 | 100 |
| S979 | kf4/Jκ4 (1) | J558.6/DFL16.1/JH2 | 4.5 x 10^4^ | 3.8 x 10^-4^ | 8.4 x 10^-9^ | 410 | 139 |
| S1069 | kf4/Jκ4 (1) | J558.54.148/DSP2.5/JH2 | 5.3 x 10^4^ | 3.3 x 10^-4^ | 6.2 x 10^-9^ | 364 | 96 |
| D5O 138 | kf4/Jκ4 (1) | J558.54.148/DSP2.2/JH2 | 5.8 x 10^4^ | 4.4 x 10^-4^ | 7.6 x 10^-9^ | 568 | 86 |
| S512 | kf4/Jκ4 (2) | J558.54.148/DSP2.5/JH2 | 6.0 x 10^4^ | 4.5 x 10^-4^ | 7.5 x 10^-9^ | 430 | 83 |
| D5O 132 | kf4/Jκ4 (3) | J558.17/DSP2.5/JH2 | 5.2 x 10^4^ | 5.1 x 10^-4^ | 9.8 x 10^-9^ | 400 | 63 |
| D5O 42 | kf4/Jκ4 (1) | VH7183.a30.50/DSP2.5/JH2 | 6.4 x 10^4^ | 4.6 x 10^-4^ | 7.2 x 10^-9^ | 308 | 74 |
| S745 | kf4/Jκ4 (1) | VHQ52.a27.79/DSP2.5/JH2 | 7.8 x 10^4^ | 8.5 x 10^-4^ | 1.1 x 10^-8^ | 457 | 134 |
| S618 | kf4/Jκ4 (1) | VH9.12/DFL16.1/JH2 | 4.8 x 10^4^ | 4.9 x 10^-4^ | 1.0 x 10^-8^ | 435 | 78 |
| D5O 96 | kf4/Jκ2 (1) | J558.6/DFL16.1/JH2 | 1.1 x 10^4^ | 2.8 x 10^-4^ | 2.6 x 10^-9^ | 289 | 125 |
| S972 | kf4/Jκ2 (1) | J558.6/DFL16.1/JH3 | 6.0 x 10^4^ | 5.7 x 10^-4^ | 9.5 x 10^-9^ | 544 | 150 |
| S915 | kf4/Jκ2 (2) | J558.54.148/DSP2.5/JH2 | 5.1 x 10^4^ | 4.5 x 10^-4^ | 8.9 x 10^-9^ | 143 | 39 |
| S919 | kf4/Jκ2 (1) | VH7183.a30.50/DSP2.5/JH2 | 7.17 x 10^4^ | 9.4 x 10^-4^ | 1.3 x 10^-8^ | 455 | 139 |
| S642 | kf4/Jκ2 (1) | VHQ52.a24.72/DSP2.5/JH2 | 3.5 x 10^4^ | 3.4 x 10^-4^ | 9.7 x 10^-9^ | 275 | 92 |
| S119 | cw9/Jκ4 | J558.12/DSP2.5/JH2 (2) | 6.8 x 10^4^ | 1.1 x 10^-4^ | 1.7 x 10^-9^ | 173 | 34 |
| S520 | cw9/Jκ4 | J558.12/DSP2.5/JH3 (1) | 6.1 x 10^4^ | 6.2 x 10^-4^ | 1.0 x 10^-8^ | 508 | 96 |
| D5O T120 53 | cw9/Jκ4 | J558.12/DSP2.5/JH3 (2) | 6.8 x 10^4^ | 7.3 x 10^-4^ | 1.1 x 10^-8^ | 489 | 109 |
| TT-FC 6 | cw9/Jκ4 | J558.12/DSP2.5/JH3 (3) | 7.6 x 10^4^ | 7.3 x 10^-4^ | 9.6 x 10^-9^ | 536 | 112 |
| TT-FC 35 | cw9/Jκ4 | J558.12/DSP2.5/JH3 (4) | 8.2 x 10^4^ | 5.8 x 10^-4^ | 7.1 x 10^-9^ | 475 | 101 |
| S521 | cw9/Jκ4 | J558.12/DSP2.5/JH4 | 7.2 x 10^4^ | 6.0 x 10^-4^ | 8.3 x 10^-9^ | 549 | 111 |
| S951 | ce9/Jκ1 | VH7183.a15.24/DFL16.1/JH2 | 8.0 x 10^4^ | 1.4 x 10^-3^ | 1.8 x 10^-8^ | 485 | 99 |
| S68 | ce9/Jκ1 | VH7183.a15.24/DSP2.3/JH2 | 8.2 x 10^4^ | 2.1 x 10^-3^ | 1.9 x 10^-8^ | 215 | 76 |
| S917 | ce9/Jκ1 | VH7183.a15.24/DSP2.10/JH4 | 2.4 x 10^5^ | 4.8 x 10^-3^ | 5.1 x 10^-9^ | 390 | 111 |
| D5O 85 | ce9/Jκ1 | VH7183.a19.31/DFL16.1/JH4 | 7.1 x 10^4^ | 1.4 x 10^-3^ | 2.0 x 10^-8^ | 394 | 114 |
| S779 | 19-15/Jκ5 | VH7183.a10.15/DSP2.5/JH3 | 6.8 x 10^4^ | 6.0 x 10^-4^ | 8.8 x 10^-9^ | 499 | 162 |

^1^Numbers in parentheses indicate clonotypes as shown in Table 3.
