# Supplementary material for: Diversity of the Antibody Response to Tetanus Toxoid: Comparison of Hybridoma Library to Phage Display Library
Source: PLoS One. 2014 Sep 30;9(9):e106699. doi: 10.1371/journal.pone.0106699 (PMC4182348; doi:10.1371/journal.pone.0106699)
Supplement: Table S3 — PCR Primers for the Amplification of Mouse Vκ and VH Gene Repertoires for Phage Display Library. (DOC) [file pone.0106699.s004.doc]

Supplementary Table 3. PCR Primers for the Amplification of Mouse Vκ and V_H_ Gene Repertoires for Phage Display Library

| **Vκ Primers^1^** | |
| --- | --- |
| Vκfr1A-TT | ATAT**GTGCAC**TTGACRTCMAGATRAYCCAGWCTMCA |
| Vκfr1B-TT | ATAT**GTGCAC**TTRAMATTKTGCTGACYCARTYTCC |
| Vκfr1C-TT | ATAT**GTGCAC**TTGATRYTKTGATGACCCAAACTCCA |
| Vκfr1D-TT | ATAT**GTGCAC**TTSRAAWTSTTCTCWYMCAGTCTCC |
| Vκfr1E-TT | ATAT**GTGCAC**TTRRCRTTSWGATGWCACAGTCKCCA |
| Vκfr1F-TT | ATAT**GTGCAC**TTGATATTGTGATRACKCAGGMTRMA |
| Vκfr1G-TT | ATAT**GTGCAC**TTRRYATTGTGATGACCCARWCWC |
| Rev Kappa AscI | AATT**GGCGCGCC**TTATTAACACTCATTCCTGTTGAAGCTCTTG |
| **V_H_ primary primers^2^** | |
| VH1 no RE | GAGGTGCAGCTTCAGGAGTCAGG |
| VH2 no RE | GATGTGCAGCTTCAGGAGTCRGG |
| VH3 no RE | CAGGTGCAGCTGAAGSAGTCAGG |
| VH4/6 no RE | GAGGTYCAGCTGCARCARTCTGG |
| VH5/9 no RE | CAGGTYCARCTGCAGCAGYCTGG |
| VH7 no RE | GARGTGAAGCTGGTGGARTCTGG |
| VH8 no RE | GAGGTTCAGCTTCAGCAGTCTGG |
| VH10 no RE | GAAGTGCAGCTGKTGGAGWCTGG |
| VH11 no RE | CAGATCCAGTTGCTGCAGTCTGG |
| Rev Gamma 1/2 no RE | CAATTTTCTTGTCCACCKYGGTSYTGCTGGCYGG |
| **V_H_ nested primers^2^** | |
| VH1 SfiI | GTCCTCGCAACTGC**GGCCCAGCCGGCC**ATGGCCGAGGTGCAGCTTCAGGAGTCAGG |
| VH2 SfiI | GTCCTCGCAACTGC**GGCCCAGCCGGCC**ATGGCCGATGTGCAGCTTCAGGAGTCRGG |
| VH3 SfiI | GTCCTCGCAACTGC**GGCCCAGCCGGCC**ATGGCCCAGGTGCAGCTGAAGSAGTCAGG |
| VH4/6 SfiI | GTCCTCGCAACTGC**GGCCCAGCCGGCC**ATGGCCGAGGTYCAGCTGCARCARTCTGG |
| VH5/9 SfiI | GTCCTCGCAACTGC**GGCCCAGCCGGCC**ATGGCCCAGGTYCARCTGCAGCAGYCTGG |
| VH7 SfiI | GTCCTCGCAACTGC**GGCCCAGCCGGCC**ATGGCCGARGTGAAGCTGGTGGARTCTGG |
| VH8 SfiI | GTCCTCGCAACTGC**GGCCCAGCCGGCC**ATGGCCGAGGTTCAGCTTCAGCAGTCTGG |
| VH10 SfiI | GTCCTCGCAACTGC**GGCCCAGCCGGCC**ATGGCCGAAGTGCAGCTGKTGGAGWCTGG |
| VH11 SfiI | GTCCTCGCAACTGC**GGCCCAGCCGGCC**ATGGCCCAGATCCAGTTGCTGCAGTCTGG |
| Rev Gamma 1/2 NotI | GAGTCATTCTCGACT**GCGGCCGC**AATTTTCTTGTCCACCKYGGTSYTGCTGGCYGG |

Restriction sites are in bold font (ApaLI/Vκ forward primers, AscI/Vκ reverse primer, SfiI/VH nested and NotI /V_H_ Rev Gamma 1/2 nested primer).

^1^Adapted from Kettleborough, *et al.*

^2^Forward primers from Amersdorfer, *et al.*
